# Association between Cystatin C and Cardiac Function in Acute Myocardial Infarction Patients: A Real-World Analysis

**DOI:** 10.1155/2022/7267937

**Published:** 2022-04-23

**Authors:** Bowen Lou, Yongbai Luo, Haoxuan Zhang, Haoyu Wu, Gulinigaer Tuerhong Jiang, Hui Liu, Kejia Kan, Xiang Hao, Lizhe Sun, Zuyi Yuan, Jianqing She

**Affiliations:** ^1^Cardiovascular Department, First Affiliated Hospital of Xi'an Jiaotong University, 277 West Yanta Road, Xi'an 710061, China; ^2^Key Laboratory of Environment and Genes Related to Diseases, Ministry of Education, Xi'an 710061, China; ^3^Southwest Jiaotong University, Chengdu, 611756 Sichuan, China; ^4^Biological Science, Georgia State University, GA 30303, USA; ^5^Biobank, First Affiliated Hospital of Xi'an Jiaotong University, Xi'an, 710061 Shaanxi, China; ^6^Department of Surgery, Universitätsmedizin Mannheim, Medical Faculty Mannheim, Heidelberg University, 68167 Mannheim, Germany

## Abstract

**Background:**

Acute myocardial infarction (AMI), as well as its long-term and short-term complications, is known to present with high morbidity and mortality. Cardiac function deterioration and ventricular remodelling after AMI are known to be correlated to worse long-term outcomes. However, the underlying mechanism remains elusive and there is a shortage of serum prediction markers. This study investigates the relationship between in-hospital Cystatin C (CysC) and cardiac function and subsequent prognosis among AMI patients. *Research Design and Methods*. We measured admission CysC and cardiac function parameters, including ejection fraction (EF) and pro-BNP value in 5956 patients diagnosed with AMI. Simple and multiregression analyses were performed to investigate the correlation between CysC and cardiac function in AMI patients. Major adverse cardiovascular events (MACE), cardiovascular, and all-cause mortality were documented, and 351 participants with high cystatin (≥1.09 mg/L) and 714 low cystatin (<1.09 mg/L) were investigated for survival analysis during a 48-month follow-up.

**Results:**

5956 patients with AMI were enrolled in the initial observational analysis, and 1065 patients of the whole cohort were included in the follow-up survival analysis. The admission CysC level was found to be significantly positively correlated to the pro-BNP level (*R* square = 0.2142, 95% CI 4758 to 5265, *p* < 0.0001) and negatively correlated to the EF value (*R* square = 0.0095, 95% CI -3.503 to -1.605, *p* < 0.0001). Kaplan-Meier survival analysis revealed significantly increased MACE incidence (HR = 2.293, 95% CI 1.400 to 3.755, *p* < 0.0001), cardiovascular mortality (HR = 3.016, 95% CI 1.694 to 5.371, *p* = 0.0002), and all-cause mortality (HR = 3.424, 95% CI 2.010 to 5.835, *p* < 0.0001) in high-admission CysC cohort with AMI at the end of 4-year follow-up.

**Conclusions:**

Admission CysC is negatively correlated with cardiac function in AMI patients and acts as a novel predictor for MACE incidence in the whole population. Further studies are needed to investigate the specific mechanism of CysC in the cardiac function deterioration among AMI patients.

## 1. Introduction

As one of the leading health-threatening diseases worldwide [1], acute myocardial infarction (AMI) is associated with substantial morbidity and mortality [2]. Despite advances in percutaneous coronary interventions (PCI) and their widespread use, the mortality rate of AMI patients, together with its complications, such as heart failure, severe arrhythmia, myocardial free wall rupture (MFWR), and cardiogenic shock (CS), remain very high [3, 4]. Especially, cardiac function deterioration and ventricular remodelling after AMI are known to be correlated with increased rehospitalization rate and worse long-term outcomes [5] and have attracted more and more attention. However, the underlying mechanism remains elusive and there is a shortage of serum prediction markers.

Cystatin C (CysC), a low-molecular-weight (13 kDa) protease inhibitor, is synthesized and released into the blood by all nucleated cells, freely filtered by kidney glomerulus and almost completely reabsorbed and metabolized by the proximal tubule, but not secreted [6]. Due to these properties, even very small changes in the glomerular filtration rate (GFR) may significantly alter serum CysC level, potentially making this basic protein a very sensitive marker of renal filtration [7]. Since it was first described in 1961 by Jorgen Clausen in human cerebrospinal fluid [8], CysC has been thoroughly investigated and is considered a promising biomarker for several diseases, including but not limited to kidney disease and nephropathy-related diabetes [9, 10], Alzheimer's disease [11], and breast cancer [12].

CysC plays pleiotropic roles in human vascular pathophysiology, particularly in regulating cathepsins S and K [13]. In vivo and in vitro studies have shown elevated levels of cathepsins and lower levels of CysC, which behaves as a potent cathepsin inhibitor—in atherosclerotic tissue [14]. Correspondingly, several studies investigated the functional role of CysC in cardiovascular disease (CVD). An observational meta-analysis showed a strong dose-dependent relation between cystatin C concentrations and CVD [14]. Rothenbacher and his team found the use of cysC based chronic kidney disease (CKD) may result in more accurate risk estimates and have better prognostic value for CVD than creatinine [15]. Additionally, in high-risk patients after ACS, CysC is a strong predictor of major adverse cardiovascular events (MACE), including death from cardiovascular causes and hospitalization for heart failure [16]. However, the evidence for the relationship between in-hospital CysC and cardiac function and subsequent long-term prognosis among AMI patients remains unclear.

In this retrospective cohort study, we investigate the relationship between admission CysC and cardiac in AMI patients. Subsequently, survival analysis was performed to investigate the effects of admission CysC levels on long-term mortality and morbidity in AMI patients.

## 2. Methods

### 2.1. Study Design and Participants

This was a single-center, retrospective cohort study. Consecutive patients admitted to the cardiology department of the First Affiliated Hospital of Xi'an Jiaotong University for AMI between January 2016 and December 2020 were enrolled. The inclusion criteria were confirmed admission diagnosis of AMI, and AMI was defined based on the universal definition criteria by the American Cardiology College [1]. The exclusion criteria were [1] severe noncardiac disease with an expected survival of less than 1 year and unwillingness to participate, [2] patients over the age of 80 years or living far away from the hospital's catchment area, and (3)extremely high CysC level (>5 mg/L). A patient could only be included once. The medical records of the patients were collected from the Biobank of the First Affiliated Hospital of Xi'an Jiaotong University, which contains deidentified data derived from raw medical records. Information about patients' present medication, vascular risk factors, and detailed medical history were obtained via questionnaires. Follow-up information was obtained via telephone and questionnaires by the general practitioner (GP). Patients' MACE, including new-onset myocardial infarction, acute heart failure and cardiac death, and cardiovascular and all-cause mortality were documented during follow-up. Written informed consent was obtained from all study participants, with ethnic committee approval at the First Affiliated Hospital of Xi'an Jiaotong University.

### 2.2. Clinical Data Collection

Detailed medical histories were screened from the patients enrolled. Patient characteristics were collected, including age, sex, disease history, and physical examination. Serum CysC levels of all patients were measured within 3 h of admission, by colloidal gold particle-enhanced colorimetric immunoassay (Nescauto GC Cystatin C, Alfresa Pharma, Osaka, Japan) with a Hitachi 7600-110 automatic analyzer. Other biochemical results were evaluated immediately after the patients' admission to the hospital. They were all collected prior to PCI. Echocardiography was performed during hospital treatment.

### 2.3. Statistical Analysis

All statistical analyses were performed by using SPSS for Mac 22.0 (SPSS Inc., Chicago, IL) or GraphPad 9.0 Prism (GraphPad Software San Diego, CA). Data were presented as frequencies or percentages for categorical variables and mean ± SD for continuous variables, unless otherwise indicated. Simple *t*-test was used to compare continuous variables which are in the normal distribution. Mann–Whitney *U* test was used to compare continuous variables which do not conform to the normal distribution. *χ*^2^ test was used to compare categorical variables. One-way ANOVA was used to compare continuous variables of three or more independent (unrelated) groups. Simple linear analysis was used for calculating the correlation between CysC and cardiac function parameters. Kaplan-Meier survival curve analysis was used to represent the proportional risk of MACE, cardiovascular, and all-cause mortality for the admission CysC values in AMI patients. A Cox proportional-hazards model was performed to provide a point estimate HR (hazard ratio) and a two-sided 95% confidence interval. Receiver-operator characteristic (ROC) curve analysis and the area under the ROC curve (AUC) were used to compare the predictive value of MACE, cardiovascular, and all-cause mortality among CysC and other indexes. A value of *p* < 0.05 was considered statistically significant.

## 3. Results

### 3.1. Study Population

**Figure 1:**
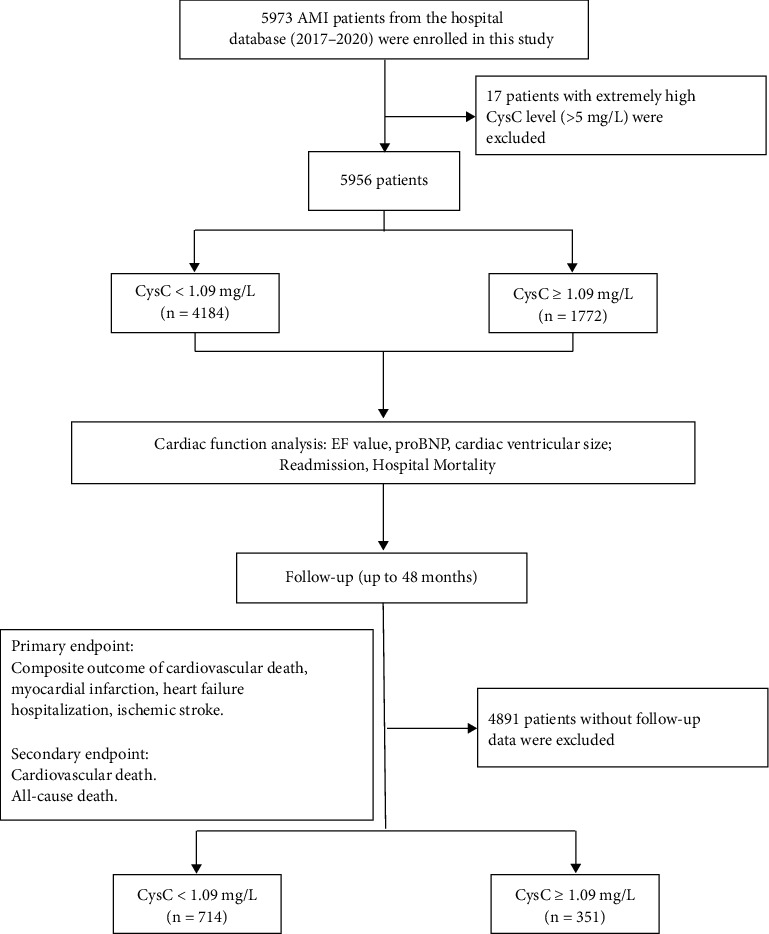
Study design, patient selection, and follow-up.

From January 2017 till December 2020, a total of 5973 AMI patients were enrolled in the study and 17 patients with extremely high CysC levels (>5 mg/L) were excluded. According to the universal definition criteria and Cutoff Finder, [17, 18] all populations were divided into the high-admission CysC cohort (1772 patients, CysC ≥ 1.09 mg/L) and low-admission CysC cohort (4184 patients, CysC < 1.09 mg/L) in the initial observational analysis, while 714 low CysC patients and 351 high CysC patients were included in the follow-up survival analysis (Figure 1). Baseline patients' characteristics are shown in Table 1, and the correlation between admission CysC and other metabolomic indexes is displayed in Table 2. The mean value of CysC was 0.76 ± 0.19 mg/L in low CysC and 1.41 ± 0.47 mg/L in the high CysC cohort. The medication was started at admission. No significant difference in blood pressure/HbA1c/TG at baseline was seen in different CysC groups in AMI patients.

### 3.2. Association between CysC and Cardiac Function in AMI patients

**Figure 2:**
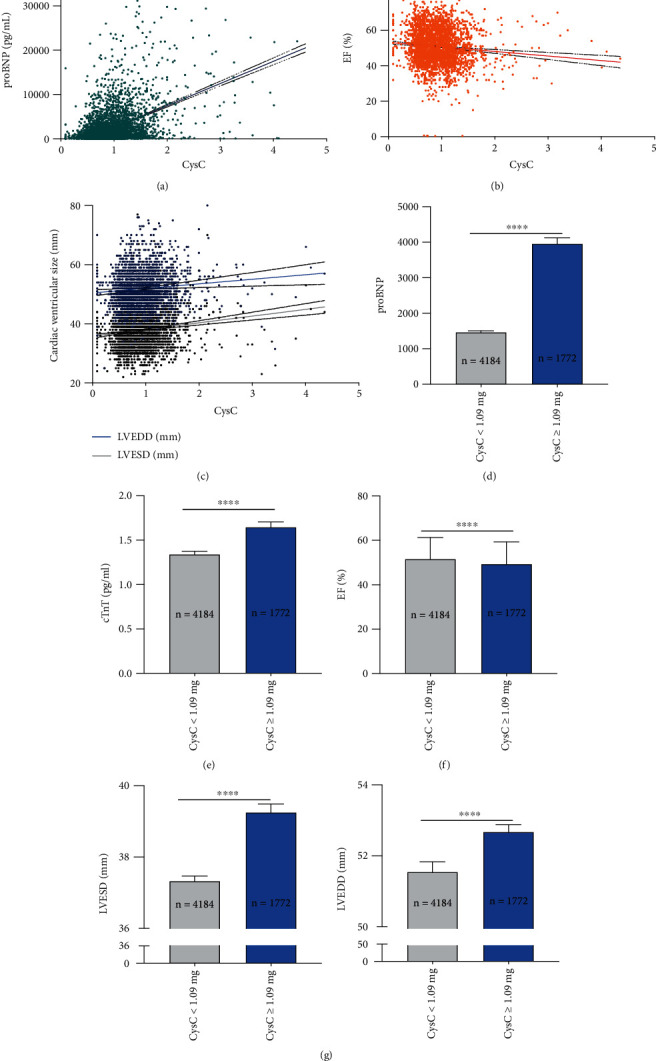
Admission CysC level was found to be significantly negatively correlated to the cardiac function in AMI patients. (a) Simple linear regression displayed a significantly positive correlation between admission CysC pro-BNP levels. (b, c) Echocardiography analysis showed a negative correlation between CysC and left ventricular EF value (b) and a positive correlation between CysC and left ventricular size, with both increased LVESD and LVEDD (c). (d–g) Subgroup analysis showed significant elevated pro-BNP value (d), elevated cTnT value (e), decreased EF value (f), and increased left ventricular size (g) in high-admission CysC cohort (CysC ≥ 1.09 mg/L) than controls (CysC < 1.09 mg/L). Data were shown in mean ± SD. For statistical analysis, Student's *t*-test was applied, ^∗∗∗∗^*p* < 0.0001. EF: ejection fraction; LVESD: left ventricular end-systolic dimension; LVEDD: left ventricular end-diastolic dimension.

To investigate the relationship between CysC and cardiac function, we utilized simple linear regression analysis. The admission CysC level was found to be significantly positively correlated to the pro-BNP level (*R* square = 0.2142, 95% CI 4758 to 5265, *p* < 0.0001) (Figure 2(a)). Echocardiography analysis showed negative correlation between CysC and left ventricular ejection fraction value (EF, *R* square = 0.0095, 95% CI -3.503 to -1.605, *p* < 0.0001) (Figure 2(b)) and positive correlation between CysC and left ventricular size, with both increased left ventricular end-systolic dimension (LVESD, *R* square = 0.0184, 95% CI 1.652 to 2.904, *p* < 0.0001) and left ventricular end-diastolic dimension (LVEDD, *R* square = 0.0028, 95% CI 0.4422 to 2.631, *p* = 0.0059) (Figure 2(c)).

Subgroup analysis further indicated that, consistently, circulating pro-BNP and cardiac troponin T (cTnT) was higher in high-admission CysC cohort than controls (pro-BNP: 1222 vs. 577.4, *p* < 0.001; cTnT 1.340 vs. 1.644, *p* < 0.001) (Figures 2(d) and 2(e)). We also found that high-admission CysC cohort displayed decreased EF value (49.31 ± 10.31 vs. 51.58 ± 9.67, *p* < 0.001) (Figure 2(f)) and increased left ventricular size evaluated by the echocardiography (Figure 2(g)).

### 3.3. Increased Hospital Mortality in High-Admission CysC Cohort with AMI

As the most frequently used risk assessment tools, the ‘Global Registry of Acute Coronary Events' (GRACE) and the ‘Can Rapid risk stratification of Unstable angina patients Suppress Adverse outcomes with Early implementation of the American College of Cardiology/American Heart Association guidelines' (CRUSADE) scores were recommended in describing the severity and mortality risk of AMI patients and management [19]. The average GRACE and CRUSADE scores were 124.5 and 21.74 in all AMI patients, respectively. Both GRACE and CRUSADE scores were significantly positively correlated to the CysC value (Fig S1). In subgroup analysis, we found GRACE and CRUSADE scores were significantly higher in high-admission CysC cohort than controls (GRACE score 133.6 vs. 121.3, *p* < 0.0001; CRUSADE score 25.78 vs. 20.18, *p* < 0.0001) (Figure 2(a)).

5038 (84.0%) AMI patients were completely reperfused with thrombolysis in myocardial infarction (TIMI) > 2 after PCI, and 918 (16.0%) AMI patients failed to reperfusion with TIMI ≤ 2 and only received medication treatment and intervention. No reflow or slow flow following PCI is independently associated with increased in-hospital mortality, malignant arrhythmias, and cardiac failure [20]. Interestingly, CysC was significantly higher in group TIMI ≤ 2 compared to group TIMI > 2 (0.93 vs. 1.13, *p* < 0.0001).

**Figure 3:**
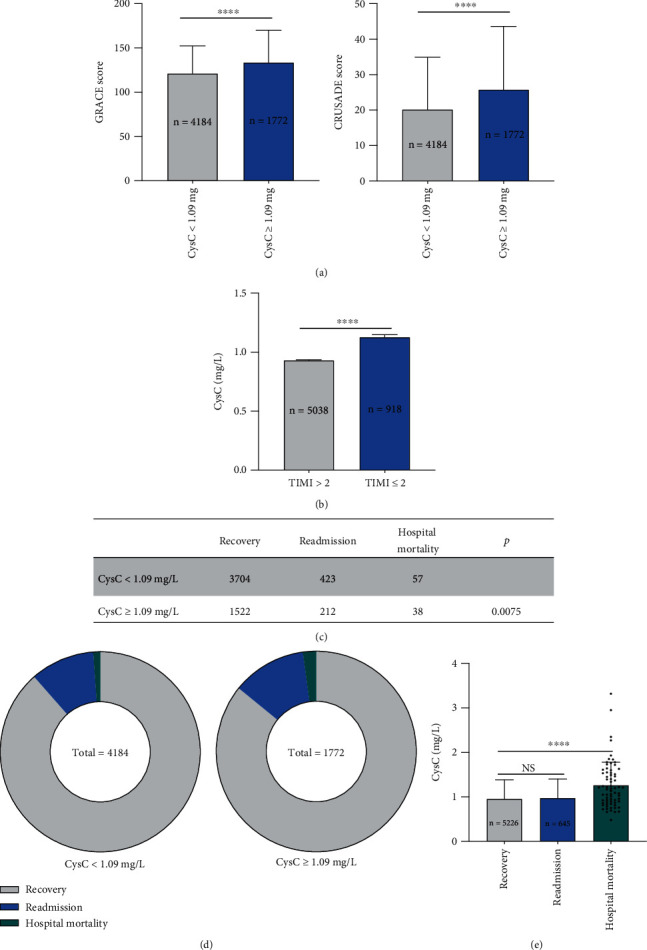
Increased hospital mortality in high-admission CysC cohort with AMI. (a) GRACE and CRUSADE score were significantly higher in high-admission CysC cohort (CysC ≥ 1.09 mg/L) than controls (CysC < 1.09 mg/L). (b) CysC was significantly higher in group TIMI ≤ 2 compared to group TIMI > 2. (c, d) High-admission CysC cohort (CysC ≥ 1.09 mg/L) showed elevated mortality rate even during hospitalization and readmission rate than controls (CysC < 1.09 mg/L). Within the high CysC cohort, 38 (2.14%) patients died for all causes during hospitalization and 212 (11.96%) for readmission. Within the low CysC cohort, 57 (1.36%) patients died for all causes during hospitalization and 423 (10.11%) for readmission. For statistical analysis, *χ*^2^ test was performed. (e) AMI patients who died during hospitalization exhibited raised admission CysC value than recovery patients, but no significant difference between readmission and recovery patients. Data were shown in mean ± SD (a, e), mean ± SEM (Bb), or as each individual dot. For statistical analysis, one-way ANOVA followed by Sidak's multiple comparison test was applied, ^∗∗∗∗^*p* < 0.0001.

Furthermore, the high-admission CysC cohort with AMI showed an elevated mortality rate during hospitalization and readmission rate than controls. Within the high CysC cohort, 38 (2.14%) patients died for all-cause during hospitalization and 212 (11.96%) had readmission to hospital. Within low CysC cohort, 57 (1.36%) patients died for all-cause during hospitalization and 423 (10.11%) readmission(Figures 3(a) and 3(b)). In addition, AMI patients who died during hospitalization exhibited raised admission CysC value than recovery patients (1.26 vs. 0.96, *p* < 0.0001), but there is no significant difference between readmission and recovery patients (0.97 vs. 0.96, *p* = 0.3133) (Figure 3(c)).

### 3.4. Increased MACE, Cardiovascular, and All-Cause Mortality Incidence in High-Admission CysC Cohort with AMI

At the end of the 48-month follow-up, within high CysC cohort, 38 (10.83%) MACE events occurred, 31 (8.83%) died for cardiac cause, and 38 (10.83%) patients for all-cause. Within low CysC cohort, 32 (4.48%) MACE events occurred, 20 (2.80%) died for cardiac cause, and 22 (3.08%) patients for all-cause.

**Figure 4:**
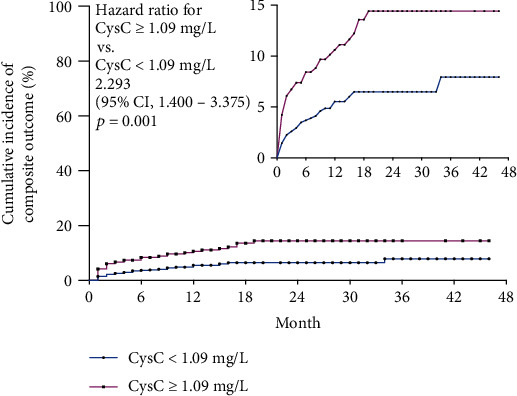
Kaplan-Meier survival analysis revealed significantly increased MACE incidence in high-admission CysC cohort with AMI at the end of 4-year follow-up. Kaplan-Meier survival analysis revealed high-admission CysC cohort (CysC ≥ 1.09 mg/L) displayed significantly increased MACE incidence (HR = 2.293, 95% CI 1.400 to 3.755, *p* < 0.0001) than controls (CysC < 1.09 mg/L).

**Figure 5:**
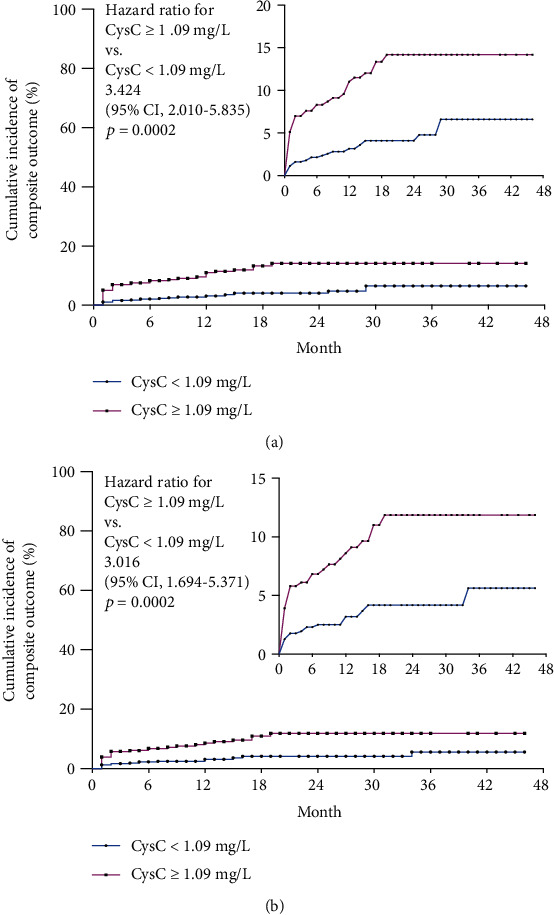
Kaplan-Meier survival analysis revealed significantly increased all/cardiovascular mortality in high-admission CysC cohort with AMI at the end of the 4-year follow-up. (a) Kaplan-Meier survival analysis revealed high-admission CysC cohort (CysC ≥ 1.09 mg/L) displayed significantly increased cardiovascular mortality (HR = 3.016, 95% CI 1.694 to 5.371, *p* = 0.0002) than controls (CysC < 1.09 mg/L). (b) Kaplan-Meier survival analysis revealed high-admission CysC cohort (CysC ≥ 1.09 mg/L) displayed significantly increased and all-cause mortality (HR = 3.424, 95% CI 2.010 to 5.835, *p* < 0.0001) than controls (CysC < 1.09 mg/L).

Kaplan–Meier survival analysis was utilized to evaluate the survival curve between two cohorts. Similarly, high-admission CysC cohort displayed significantly increased MACE incidence (HR = 2.293, 95% CI 1.400 to 3.755, *p* < 0.0001) (Figure 4), cardiovascular mortality (HR = 3.016, 95% CI 1.694 to 5.371, *p* = 0.0002), and all-cause mortality (HR = 3.424, 95% CI 2.010 to 5.835, *p* < 0.0001) as compared to controls (Figure 5).

**Figure 6:**
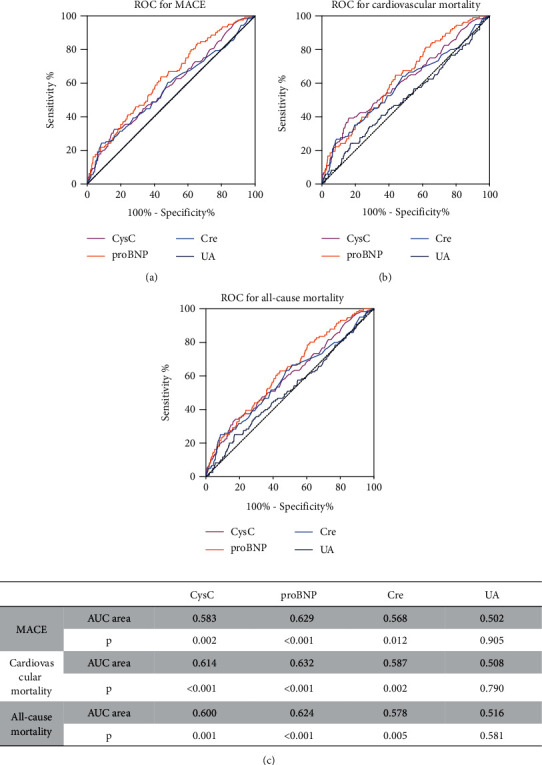
ROC for predicting MACE, cardiovascular, and all-cause mortality among CysC, pro-BNP, Cre, and UA. Accuracies of CysC, pro-BNP, Cre, and UA for predicting MACE (a), cardiovascular mortality (b), and all-cause mortality(c) presented as areas under the receiver operating characteristic curves, individually. ROC: receiver-operator characteristic; AUC: area under the receiver operating characteristic curve.

Receiver operating characteristic curves were generated, and AUCs were calculated to estimate the predicted values of different biomarkers. The performance of CysC, pro-BNP, uric acid (UA), and creatine (Cre) in predicting MACE, cardiovascular, and all-cause mortality, and MACE was illustrated in Figure 6. CysC showed significant and similar predictive accuracy as compared to pro-BNP. Cre also exhibited a significant predicting value while UA showed no difference in MACE and mortality prediction.

## 4. Discussion

In this single-center, retrospective, real-world, population-based study, we investigate the relationship between in-hospital Cystatin C (CysC) and cardiac function and subsequent prognosis among AMI patients. Serum CysC is found to be associated with cardiac function deterioration in patients with AMI. Moreover, high-admission serum CysC level exhibits high incidence of MACE as well as cardiovascular and all-cause mortality rate in AMI patients during 4-year follow-up.

The important implication of the present study is that CysC is identified as a biomarker for cardiac function in AMI patients. Several previous studies investigated the relationship between CysC and heart failure incidents. Via 4 community-based cohorts with 12.5 years of follow-up, Navin Suthahar and their team found CysC was strongly and similarly associated with HF in both sexes [21], as these biomarkers reflect distinct pathophysiological processes [22], and the elevation may indicate cardiovascular or systemic derangement early in the time course of HF progression [23]. Additionally, our result indicated that CysC might be eligible as a potential serum predictor for heart failure in the population after acute myocardial infarction.

Notably, the major outcomes of this study show increased MACE incidence, cardiovascular, and all-cause mortality in the high CysC cohort with AMI at the end of 4-year follow-up, indicating that CysC level is a potential independent predictor for cardiac prognosis after AMI. Increasing shreds of evidence have shown that higher CysC is associated with higher cardiovascular risk and mortality rate in patients with non-ST elevated acute coronary syndrome [24], and serum creatinine to cystatin C ratio is associated with major adverse cardiovascular events in patients with obstructive coronary artery disease [25]. In AMI patients, an increased admission CysC level was associated with a higher risk of in-hospital and 1-month death [26]. Besides, circulating cystatin C level on the 12th–14th day after hospital admission predicted the adverse cardiovascular outcome in patients with STEMI [27]. Through a 4-year follow-up study, we further proved that CysC can be included in the risk stratification model to guide the treatment of high-risk AMI patients.

Several potential mechanisms may account for the prognostic importance of CysC in AMI patients. First, abnormal CysC value can identify early patients with renal insufficiency before circulating creatinine, which could be linked to atherosclerosis, vascular complications, and increased cardiovascular events [28]. Second, CysC may play an important role in regulating cardiac inflammatory responses [29], contributing to the development of no-reflow and the increased risk of death. Besides, CysC elevation may damage the cardiovascular system by affecting lipid peroxidation, coagulation function, and smooth muscle cell and endothelial cell function [30], and finally, facilitate the vulnerability of atherosclerotic plaque CysC [31].

However, there are several potential limitations in the current work: first, this study is limited in its single-center, retrospective, and observational nature. A future multicenter prospective study with a larger number of patients and a longer follow-up is required. Also, several parameters, including patient age were not balanced between the high and low CysC cohort. As age might also be a prognostic factor in AMI patients, improving risk stratification independently of age and kidney function would be considered in the further study.

In conclusion, through this retrospective cohort study, we have found that admission CysC is negatively correlated with cardiac function in AMI patients and acts as a novel predictor for MACE incidence in the whole population. Further studies are needed to investigate the specific mechanism of CysC during the cardiac function deterioration of AMI patients.

## Figures and Tables

**Table 1:** Distribution of demographic and clinical characteristics according to CysC levels.

	CysC < 1.09 mg/L (*n* = 4184)	CysC ≥ 1.09 mg/L (*n* = 1722)	*p* value
CysC (mg/L)	0.76 ± 0.19	1.41 ± 0.47	<0.001
Age (years)	60.67 ± 11.68	65.79 ± 12.16	<0.001
Female sex (%)	799 (19.9)	368 (20.7)	0.434
SBP (mmHg)	123.50 ± 21.64	122.62 ± 23.45	0.168
DBP (mmHg)	77.96 ± 14.83	75.90 ± 14.82	0.182
EF (%)	51.58 ± 9.67	49.31 ± 10.31	<0.001
pro-BNP (pg/mL)	577.4	1222	<0.001
HbA1c (%)	6.28 ± 1.46	6.36 ± 1.41	0.068
TG (mmol/L)	1.49	1.59	0.013
LDL (mmol/L)	2.42 ± 0.85	2.25 ± 0.82	<0.001
HDL (mmol/L)	0.95 ± 0.23	0.92 ± 0.22	<0.001
ApoA (g/L)	1.07 ± 0.19	1.03 ± 0.19	<0.001
ApoB (g/L)	0.21 ± 0.23	0.78 ± 0.22	<0.001
ApoE (mg/L)	36.30 ± 14.14	36.01 ± 14.36	0.490
Cre (*μ*mol/L)	64.51	99.23	<0.001
UA (*μ*mol/L)	321.33	370.06	<0.001
HomoCys (*μ*mol/L)	23.17	26.23	<0.001
Ticagrelor (%)	2060 (51.2)	787 (44.4)	<0.001
Aspirin (%)	3934 (97.8)	1773 (96.1)	0.001
Furosemide (%)	1731 (43.0)	988 (58.8)	<0.001
Spirolactone (%)	1449 (36.2)	782 (44.2)	<0.001
Isosorbide mononitrate (%)	2340 (58.1)	1040 (58.7)	0.707
Diltiazem (%)	198 (4.9)	78 (4.4)	0.422
Nifedipine (%)	399 (9.9)	262 (14.7)	<0.001
Metoprolol (%)	3327 (82.7)	1377 (77.7)	<0.001

Data were shown in mean ± SD, median ,or *n* (%). SBP: systolic blood pressure; DBP: diastolic blood pressure; HbA1c: hemoglobin A1c; TG: triglyceride; LDL: low-density lipoprotein; HDL: high-density lipoprotein; ApoA: apolipoprotein A; ApoB: apolipoprotein B; ApoE: apolipoprotein E; Cre: creatinine; UA: uric acid; HomoCys: homocysteine.

**Table 2:** Correlation between admission CysC and other metabolomic indexes.

	pro-BNP	EF	HbA1c	TC	TG	LDL	HDL	Cre	UA	HomoCys
Pearson correlation coefficient	0.2142	0.0010	0.009	-0.032	-0.022	-0.027	-0.013	0.270	0.103	0.023
*p* value	<0.0001	<0.0001	0.515	0.017	0.097	0.049	0.350	<0.0001	<0.0001	0.128
*n*	5793	5787	5193	5671	5456	5455	5456	5793	5793	4451

HbA1c: hemoglobin A1c; TC: total cholesterol; TG: triglyceride; LDL: low-density lipoprotein; HDL: high-density lipoprotein; Cre: creatinine; UA: uric acid; HomoCys: homocysteine.

## Data Availability

The authors confirm that the data supporting the findings of this study are available from the corresponding author JS upon reasonable request.

## Supplementary Materials

Supplementary Materials Figure S1: simple linear regression displayed a significantly positive correlation between admission CysC and GRACE/CRUSADE score.

## References

[1] DeFilippis A. P., Chapman A. R., Mills N. L. (2019). Assessment and treatment of patients with type 2 myocardial infarction and acute nonischemic myocardial injury. *Circulation*.

[2] Dhruva S. S., Ross J. S., Mortazavi B. J. (2020). Association of use of an intravascular microaxial left ventricular assist device vs intra-aortic balloon pump with in-hospital mortality and major bleeding among patients with acute myocardial infarction complicated by cardiogenic shock. *Journal of the American Medical Association*.

[3] Lou B., Luo Y., Hao X. (2019). Clinical characteristics and protective factors in patients with acute myocardial infarction undergoing in-hospital myocardial free wall rupture: a single-center, retrospective analysis. *Journal of Investigative Medicine*.

[4] Schrage B., Ibrahim K., Loehn T. (2019). Impella support for acute myocardial infarction complicated by cardiogenic shock. *Circulation*.

[5] Lewicki L., Fijalkowska M., Karwowski M. (2021). The non-invasive evaluation of heart function in patients with an acute myocardial infarction: the role of impedance cardiography. *Cardiology Journal*.

[6] Ferguson T. W., Komenda P., Tangri N. (2015). Cystatin C as a biomarker for estimating glomerular filtration rate. *Current Opinion in Nephrology and Hypertension*.

[7] Seronie-Vivien S., Delanaye P., Pieroni L., Mariat C., Froissart M. (2008). Cystatin C: current position and future prospects. *Clinical Chemistry and Laboratory Medicine*.

[8] Grubb A., Lofberg H. (1982). Human gamma-trace, a basic microprotein: amino acid sequence and presence in the adenohypophysis. *Proceedings of the National Academy of Sciences of the United States of America*.

[9] Park M. Y., Choi S. J., Kim J. K., Hwang S. D., Lee Y. W. (2013). Urinary cystatin C levels as a diagnostic and prognostic biomarker in patients with acute kidney injury. *Nephrology*.

[10] Perkins B. A., Nelson R. G., Ostrander B. E. (2005). Detection of renal function decline in patients with diabetes and normal or elevated GFR by serial measurements of serum cystatin C concentration: results of a 4-year follow-up study. *Journal of the American Society of Nephrology*.

[11] Levy E., Sastre M., Kumar A. (2001). Codeposition of cystatin C with amyloid-beta protein in the brain of Alzheimer disease patients. *Journal of Neuropathology and Experimental Neurology*.

[12] Leto G., Sepporta M. V. (2020). The potential of cystatin C as a predictive biomarker in breast cancer. *Expert Review of Anticancer Therapy*.

[13] Shi G. P., Sukhova G. K., Grubb A. (1999). Cystatin C deficiency in human atherosclerosis and aortic aneurysms. *The Journal of Clinical Investigation*.

[14] van der Laan S. W., Fall T., Soumare A. (2016). Cystatin C and cardiovascular disease: a Mendelian randomization study. *Journal of the American College of Cardiology*.

[15] Rothenbacher D., Rehm M., Iacoviello L. (2020). Contribution of cystatin C- and creatinine-based definitions of chronic kidney disease to cardiovascular risk assessment in 20 population-based and 3 disease cohorts: the BiomarCaRE project. *BMC Medicine*.

[16] Correa S., Morrow D. A., Braunwald E. (2018). Cystatin C for risk stratification in patients after an acute coronary syndrome. *Journal of the American Heart Association*.

[17] Reinhard M., Erlandsen E. J., Randers E. (2009). Biological variation of cystatin C and creatinine. *Scandinavian Journal of Clinical and Laboratory Investigation*.

[18] Guo S., Xue Y., He Q. (2017). Preoperative serum cystatin-C as a potential biomarker for prognosis of renal cell carcinoma. *PLoS One*.

[19] Tscherny K., Kienbacher C., Fuhrmann V. (2020). Risk stratification in acute coronary syndrome: evaluation of the GRACE and CRUSADE scores in the setting of a tertiary care centre. *International Journal of Clinical Practice*.

[20] Ito H. (2006). No-reflow phenomenon and prognosis in patients with acute myocardial infarction. *Nature Clinical Practice. Cardiovascular Medicine*.

[21] Suthahar N., Lau E. S., Blaha M. J. (2020). Sex-specific associations of cardiovascular risk factors and biomarkers with incident heart failure. *Journal of the American College of Cardiology*.

[22] Vasan R. S. (2006). Biomarkers of cardiovascular Disease. *Circulation*.

[23] Ibrahim N. E., Januzzi J. L. (2018). Established and emerging roles of biomarkers in heart failure. *Circulation Research*.

[24] Jernberg T., Lindahl B., James S., Larsson A., Hansson L. O., Wallentin L. (2004). Cystatin C. *Circulation*.

[25] Lu Y. W., Tsai Y. L., Chou R. H. (2021). Serum creatinine to cystatin C ratio is associated with major adverse cardiovascular events in patients with obstructive coronary artery disease. *Nutrition, Metabolism, and Cardiovascular Diseases*.

[26] Fouad M., Boraie M. (2013). Cystatin C as an early marker of acute kidney injury and predictor of mortality in the intensive care unit after acute myocardial infarction. *Arab Journal of Nephrology and Transplantation*.

[27] Barbarash O. L., Bykova I. S., Kashtalap V. V. (2017). Serum neutrophil gelatinase-associated lipocalin has an advantage over serum cystatin C and glomerular filtration rate in prediction of adverse cardiovascular outcome in patients with ST-segment elevation myocardial infarction. *BMC Cardiovascular Disorders*.

[28] Machino-Ohtsuka T., Seo Y., Ishizu T. (2010). Combined assessment of carotid vulnerable plaque, renal insufficiency, eosinophilia, and hs-CRP for predicting risky aortic plaque of cholesterol crystal embolism. *Circulation Journal*.

[29] Salgado J. V., Souza F. L., Salgado B. J. (2013). How to understand the association between cystatin C levels and cardiovascular disease: imbalance, counterbalance, or consequence?. *Journal of Cardiology*.

[30] Ballew S. H., Matsushita K. (2018). Cardiovascular risk prediction in CKD. *Seminars in Nephrology*.

[31] Antoniadis A. P., Chatzizisis Y. S., Giannoglou G. D. (2008). Pathogenetic mechanisms of coronary ectasia. *International Journal of Cardiology*.

