# Supplementary material for: Association between Cystatin C and Cardiac Function in Acute Myocardial Infarction Patients: A Real-World Analysis
Source: Dis Markers. 2022 Apr 23;2022:7267937. doi: 10.1155/2022/7267937 (PMC9056268; doi:10.1155/2022/7267937)
Supplement: Supplementary Materials — Figure S1: simple linear regression displayed a significantly positive correlation between admission CysC and GRACE/CRUSADE score. [file 7267937.f1.pptx]

## Slide 1
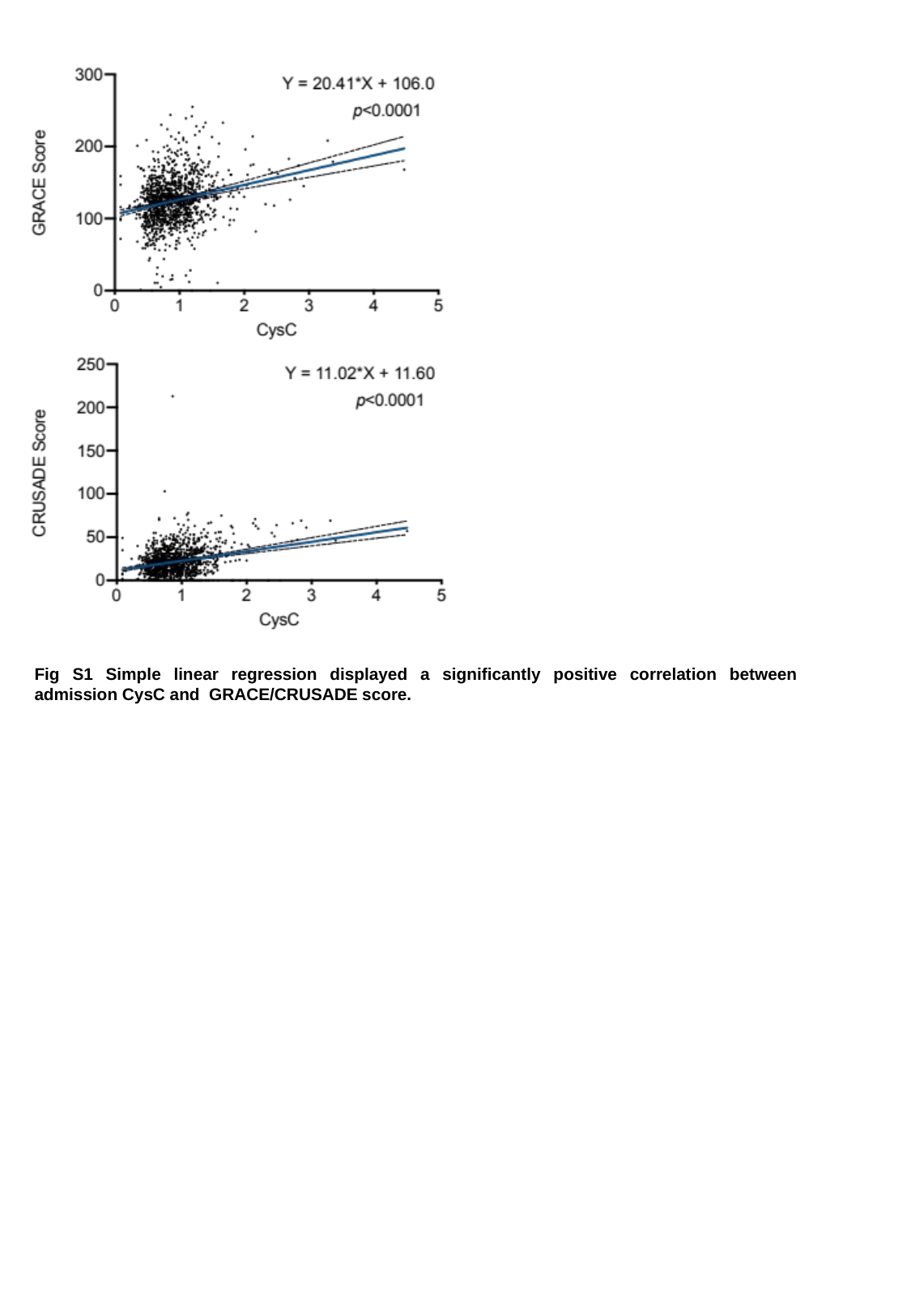

Fig S1 Simple linear regression displayed a significantly positive correlation between admission CysC and GRACE/CRUSADE score.
